# Revealing the core-shell interactions of a giant strain relaxor ferroelectric 0.75Bi_1/2_Na_1/2_TiO_3_-0.25SrTiO_3_

**DOI:** 10.1038/srep36910

**Published:** 2016-11-14

**Authors:** Na Liu, Matias Acosta, Shuai Wang, Bai-Xiang Xu, Robert W. Stark, Christian Dietz

**Affiliations:** 1 Institute of Materials Science and Center of Smart Interfaces, Physics of Surfaces, Technische Universität Darmstadt, Alarich-Weiss-Str. 10, 64287 Darmstadt, Germany; 2 Insititute of Materials Science, Nichtmetallische-Anorganische Werkstoffe, Technische Universität Darmstadt, Alarich-Weiss-Str. 2, 64287 Darmstadt, Germany; 3 Insititute of Materials Science, Mechanik funktionaler Materialien, Technische Universität Darmstadt, Jovanka-Bontschits-Str. 2, 64287 Darmstadt, Germany

## Abstract

Lead-free relaxor ferroelectrics that feature a core-shell microstructure provide an excellent electromechanical response. They even have the potential to replace the environmentally hazardous lead-zirconia-titanate (PZT) in large strain actuation applications. Although the dielectric properties of core-shell ceramics have been extensively investigated, their piezoelectric properties are not yet well understood. To unravel the interfacial core-shell interaction, we studied the relaxation behaviour of field-induced ferroelectric domains in 0.75Bi_1/2_Na_1/2_TiO_3_-0.25SrTiO_3_ (BNT-25ST), as a typical core-shell bulk material, using a piezoresponse force microscope. We found that after poling, lateral domains emerged at the core-shell interface and propagated to the shell region. Phase field simulations showed that the increased electrical potential beneath the core is responsible for the in-plane domain evolution. Our results imply that the field-induced domains act as pivotal points at the coherent heterophase core-shell interface, reinforcing the phase transition in the non-polar shell and thus promoting the giant strain.

Concerns about the harmful effects of lead-containing piezoelectrics on the environment as well as human and animal health drive the development of lead-free relaxor ferroelectrics (relaxors)^1^,^2^,^3^,^4^,^5^,^6^,^7^. Three perovskite systems based on BaTiO_3_, (K,Na)NbO_3_ and Bi_**1/2**_Na_**1/2**_TiO_3_ have been identified as competitive materials that feature an electric field-induced giant strain akin to or even surpassing the strain of PZT-based materials^8^,^9^,^10^,^11^. Generally, their large strain response is associated with a reversible field-induced phase transition from a macroscopic ergodic relaxor state to a ferroelectric state with a long-range order^12^,^13^, as recently demonstrated by piezoresponse force microscopy (PFM)^14^. Nevertheless, the technological use of these materials thus far remains limited, mainly because of their large hysteresis and the large electric fields required to trigger the phase transition^10^,^15^. Current research activities have, however, led to novel systems with increasingly large strains albeit at too large electric fields^16^,^17^,^18^. Liu and Tan^17^ demonstrated that in a complex Bi-based system ((Bi_1/2_(Na_0.84_K_0.16_)_1/2_)_0.96_Sr_0.04_)(Ti_1−*x*_Nb_*x*_)O_3_, it was feasible to obtain an electrostrain under unipolar fields as large as 0.65% at 5 kV/mm, corresponding to a piezoelectric coefficient *d*_33_^*^ = 1300 pm/V. The high strain was attributed to the phase transitions between an ergodic relaxor state with coexisting rhombohedral and tetragonal phases a ferroelectric rhombohedral phase. In addition to these advancements, it has been shown that the strain mechanisms behind large electrostrain materials can be highly complex, and not only piezoelectricity but also rotostriction might play a crucial role^19^. Recent studies highlighted that the strain state^20^ and intergranular constraints could greatly influence the phase transitions leading to a giant strain, thus paving the way to new potential developmental routes^19^,^21^.

The synthesis of a bulk core-shell material has been one of the recent approaches for improving the electromechanical response while reducing the hysteresis^1^. In BNT-25ST, which is a typical representative for a relaxor core-shell material, the formation of a core-shell structure is driven by the low diffusion rate of Sr during the sintering process, which leads to nanometre-sized, Sr^2^^+^ depleted regions that eventually turn into rhombohedral core regions. The core-shell structure is a non-equilibrium metastable state that can be removed gradually by extending the sintering time. Thus, the core density and average grain size can be engineered. Both parameters are crucial factors for the macroscopic electromechanical performance of the material^3^. The core-shell interface between the coherent heterophases also has a considerable effect on the macroscopic properties under an electrical stimulus^12^,^22^,^23^,^24^. Nevertheless, the physical mechanism of the domain evolution at the core-shell interface and the respective structure-property relationship remain unexplored for both dielectric and piezoelectric materials.

Piezoresponse force microscopy is a well-established tool for directly and nondestructively mapping the domain structure and evolution of field-induced polarisations on the surfaces of ferroelectrics, with nanometre precision^25^,^26^. Both the in-plane and out-of-plane polarisation can be mapped on the sample surface^25^,^27^. The domain distribution and evolution strongly depend on the variability of the polarisation beneath the surface. Here, phase field simulations have emerged as attractive computational tools for predicting the evolution mechanisms of induced domains in bulk ferroelectrics^28^,^29^,^30^. Based on the random field theory, a generic phase field model^31^, implemented by a finite element calculation, was recently developed for the simulation of the temporal and spatial evolution of the polarisation in relaxors. Thus, theoretical predictions of the domain evolution in the bulk can be combined with experimental observations on the sample surface. Here we propose a model for the local core-shell configuration of BNT-25ST. At room temperature, BNT-25ST features a large normalized strain *d*_33_^*^ > 600 pm/V under an electric field of 4 kV/mm^2^,^3^,^32^. The virgin domain structure, as well as its field-induced switching and subsequent domain evolution, is investigated by means of PFM. The different relaxor states between core and shell are confirmed. A responsive phase-electric field relaxor model well reflects the experimental observation of the formation of lateral domains and their evolution at the core-shell interface.

Transmission electron microscopy (TEM), X-ray diffraction (XRD) and macroscopic characterisation showed that BNT-25ST has a core-shell structure with Sr^2+^-depleted cores^1^,^32^. A typical TEM image of the microstructure of BNT-25ST exhibiting the non-ergodic core (C) and the ergodic shell (S) within an individual grain in the virgin state at room temperature is shown as inset in Fig. 1. To identify surface regions with a distinct piezoresponse in the virgin state, we first investigated an untreated sample of BNT-25ST with vector PFM. A representative core-shell structure of an individual grain is captured by the vertical phase channel (Fig. 1, note that TEM and PFM images do not show the same grain). This individual grain exhibits distinctive domain contrasts in the core (C), whereas there is only a weak contrast in the surrounding areas (S). The observation of two distinct relaxor states within the material is in good agreement with the TEM images^1^. See the Supplementary Information for different grains and their respective vector orientations of the polarisation (Supplementary Fig. S1).

In the next step, the response of the material to an external electric field (local poling) and the subsequent relaxation of the tip-induced polarisation were investigated. A surface area of 1.5 × 1.5 μm^2^ was poled within the region of interest (Supplementary Fig. S1m, dashed square). Then, the same region was analyzed 15, 45 and 90 min later. Figure 2 shows the corresponding time series of PFM images. In each row, the PFM data of one time step is summarized. The vertical phase and amplitude signals (Fig. 2a,b) indicate that a long-range ordered and downward-oriented ferroelectric domain (Fig. 2a, dark area) was successfully induced by the +10 V biased-tip 15 min after poling. However, this domain was slightly larger (~2.0 × 2.0 μm^2^) than the area that was scanned during the poling procedure (1.5 × 1.5 μm^2^). The sidewall of the tip cone around the tip apex can additionally contribute to the effective area of the electric field^33^. The tail-shaped trace at the left edge of the poled area was caused by residual charges on the tip as the tip moved away after poling.

In the vertical amplitude, a circular domain exists inside the poled area (Fig. 2b, blue arrow). This means that the tip-induced domain in the shell has partially relaxed back within the first 15 min after poling because the vertical amplitude is a measure of the out-of-plane piezoresponse. Moreover, a weak in-plane domain pattern can be observed in the lateral phase (Fig. 2c) and amplitude (Fig. 2d) images within the core-shell region. After 45 min, the induced long-range ordered vertical domain shrank down to a distinctive circular area (Fig. 2e,f), which can be identified as the non-ergodic core. The relaxation of the surrounding material can be attributed to the high ergodicity of the shell. The in-plane domain pattern was even more pronounced in the lateral phase and amplitude images 45 min after poling (Fig. 2g,h). After 90 min, the vertical phase image (Fig. 2i) did not change further, whereas the vertical amplitude signal in the core (Fig. 2j) was weaker. In contrast, the lateral phase and amplitude images remained almost unchanged after 45 min (Fig. 2g,h,k,l). A detailed study of the evolution of the field-induced vertical domain and the relaxation behaviour of the core and the shell, by means of averaged cross-sectional profiles taken in the vertical phase (Fig. 2a,e,i and Supplementary Fig. S1p), amplitude (Fig. 2b,f,j and Supplementary Fig. S1o) and the respective topography, is presented in the Supplementary Information (Supplementary Fig. S2). The compositional difference between core and shell^1^,^3^ also implies a gradual variation in the lattice constant, which can explain the small height variations that were induced by the electric field at the core (Supplementary Fig. S2a). Before poling, the interfacial region presented an equilibrium stress state that lead to a small depression at the core, which was most likely caused by different ablation rates during polishing. In an external electric field, however, the polarisation mismatch causes a stress field at the core-shell interface, which in turn leads to an immediate local volume increase after poling and the subsequent decrease during relaxation.

To better illustrate the evolution of the field-induced domain at the core-shell interface, the lateral phase piezoresponse images (Fig. 2c,g,k and Supplementary Fig. S1n) are enlarged and shown together in Fig. 3. The white lines indicate the interface between the core and its periphery. Figure 3a shows the virgin domain state, with a strong image contrast in the core but a negligible image contrast within the shell. Figure 3b reveals that the virgin lateral domain configuration completely disappeared after vertical poling. Moreover, new lateral domains were formed at preferential nucleation spots around the core-shell interface, as indicated by the blue arrows. Remarkably, 45 min after poling (Fig. 3c), these lateral domains became larger and more pronounced, in contrast to that observed previously, when they remained stable (Fig. 3d, phase image 90 min after poling). This observation indicates that lateral domains can be induced by the electrical field and start to nucleate at the core-shell interface. These freshly induced domains continue to grow laterally and propagate into the adjacent shell to compensate for the mismatch of stress and polarisation between the irreversibly induced ferroelectric state in the core and the ergodic shell, which is the driving force of the lateral domain propagation.

Under an external field, the entire virgin material can be poled. After removing the field, the core remains poled, whereas the shell reversibly relaxes to the initial state^27^,^34^. We thus propose a model for the polarisation evolution of the core-shell region in the poling experiment (Fig. 4). In the virgin state (Fig. 4a), the core is embedded in a region (azure) where randomly oriented polar nanoregions (PNRs) (encircled black arrows) characterize the material. At this stage, the core (black blue) predominantly exhibits laterally oriented domains (black arrows). To pole the material, an electric field between the AFM tip and the sample was applied. This poling process induced a downward-oriented ferroelectric domain with a long-range order (Fig. 4b, dark blue area). The AFM induced domain was not stable and shrank to a well-defined core-shell interface (white dashed frame). Then, further relaxation occurred (Fig. 4c) around the newly formed and electrically induced core-shell interface. Finally, the induced single vertical domain relaxed back to its virgin state (PNRs, encircled black arrows, Fig. 4c) because there was a random electrical potential in the ergodic shell. In the non-ergodic core, however, a single vertical domain (dark blue area) that is associated with an irreversible transition to a ferroelectric state was induced. Due to the stress and polarisation mismatch at the core-shell interface, a lateral domain nucleated and grew as compensation (brown area represents the lateral domain propagation). The actual spatial domain orientations at the core-shell interfacial area were then a complex mixture of vertical and lateral domain components (Fig. 4d).

To gain further insights into the evolution of the domain distribution after poling, a continuum phase field simulation was carried out. The configuration of a core-shell microstructure was included in the model (Fig. 5). The initial equilibrium state is shown in Fig. 5a, in which large domains are present in the non-ergodic core and a structure with small-sized domains in the ergodic shell. The corresponding potential distribution (Fig. 5b) shows a potential with small variations around zero throughout the *x*-*z* plane. The random field distribution caused by the chemical disorder leads to some hot spots with relatively high/low potential^35^. The electric field loading was then applied by a potential difference between the top and bottom boundary. Core and shell regions were fully poled under the peak potential of +10 V (Fig. 5c), with the polarisation pointing downwards. The corresponding distribution of electric potential difference indicates an almost homogeneous gradient (Fig. 5d). After the external potential is switched off, polarisation and potential distribution represent the remanent state (Fig. 5e,f): the poled polarisation state in the shell region relaxes to a random state, whereas the field-induced ferroelectric domains within the core region remain poled. The potential distribution also relaxes to zero, except for the bottom of the core-shell interface (red area). The downward polarisation of the ferroelectric domain in the non-ergodic core can thus not be compensated for and leads to the presence of a positive charge beneath the core. As a result, the potential beneath the core is higher (red area) than the one of the rest of the simulated mesh (Fig. 5f). The remanent domain configuration is symbolized as polarisation vectors (Fig. 5g), in which black frames enclose regions with predominantly laterally oriented domains (red and green arrows), which is consistent with the lateral domain phenomenon observed in the PFM experiment (*cf*. Fig. 3). The local poling induced a transition from the relaxor states (ergodic – shell, non-ergodic – core) to the ferroelectric state in both regions, which was completely reversible in the shell area but caused the core region to be permanently in the ferroelectric state. These findings and the appearance/growth of a ferroelectric domain with a polarisation direction perpendicular to the applied field into the ergodic shell are in good agreement with the results obtained by the phase field simulation based on a core-shell microstructure model. It revealed a strong gradient in the electrical potential at the interfacial area, which causes the nucleation and propagation of an in-plane polarisation.

The phase field simulation demonstrated that the observed evolution of field-induced lateral domains via PFM is due to a compensation of polarisation charges at the core-shell interface beneath the sample surface. Transferring these local observations within an individual grain to the macroscopic scale offers new insights into the performance and functionality of actuators: actuator applications require materials with large, recoverable, electric field-induced strain outputs executed at low fields^13^. Macroscopically, an actuator based on a relaxor runs from the remanent state (generally negligible) to a state where the entire material is held at the ferroelectric state by a sufficiently strong external electric field and returns back to the remanent state. Interpreting this cycle microscopically implies that the relaxed ergodic state of each shell is switched to a ferroelectric state and back. The core, in contrast, permanently remains in the ferroelectric state. Upon switching on the external field, the alignment of the “pivot points” at the interface from a polarisation direction perpendicular to the applied field to a parallel configuration facilitates the domain orientation in the shell. These pivot points originate from the residual high potential at the interface and generate domain nuclei at the adjacent shell. These nuclei aggregate, and the surrounding PNRs align with the external electric field. Consequently, the energy barrier for the completion of the phase transition in the shell is reduced. Conversely, upon switching off the external field, the pivot points reduce the mechanical stress at the interface that was established during the relaxation process between the polarised core and the arbitrarily distributed shell polarisation. The formation of the lateral polarisation thus plays the central role in the compensation of the polarisation and strain mismatches between the core and the shell during the poling and relaxation processes.

In summary, our findings provide an experimental and theoretical insight into the mechanism behind the macroscopic functionality of core-shell piezoceramics. The fact that the coherent heterophase core-shell interface plays such a crucial role in enhancing the polarisation under an electric field lets us conclude that tailoring the core-shell configuration with regard to shape, relative size, or the interface between core and shell can lead to more efficient materials as they are needed for technical applications.

## Methods

### Sample preparation

BNT-25ST was synthesized via a mixed oxide route employing Bi_2_O_3_ (99.975%), Na_2_CO_3_ (99.5%), TiO_2_ (99.9%), and SrCO_3_ (99%) (Alfa Aesar GmbH, Karlsruhe, Germany) as reagent-grade oxides and carbonates. The powders were mixed according to the 0.75Bi_1/2_Na_1/2_TiO_3_-0.25SrTiO_3_ stoichiometric formula. Complete processing details of this material can be found elsewhere^32^. The sample preparation processing for PFM measurements followed a protocol reported elsewhere^27^.

### Piezoresponse force microscopy

The PFM measurements were performed using a Cypher atomic force microscope (Asylum Research, Santa Barbara, CA). Ti/Ir- (5/20) and Ti/Pt- (5/20) coated conductive cantilevers ASYELEC-02 and AC240TM (both from Asylum Research, Santa Barbara, CA) were used. The nominal spring constants and fundamental resonance frequencies were *k* = 40 N/m and *f* = 300 kHz for the ASYELEC-02 cantilevers and *k* = 2 N/m and *f* = 70 kHz for AC240TM. For PFM imaging, the driving frequencies were set slightly below the respective contact resonance frequencies. Images were taken with a lateral resolution of 256 × 256 pixels; the tip was scanned perpendicular to the cantilever’s length axis. To perform the vertical domain poling, a +10 V *dc* voltage was applied to the tip while it scanned line-wise an area of 1.5 × 1.5 μm^2^ with a velocity of 1.1 μm/s. The tip-induced domain patterns were successively characterized, with a scan area of 3.0 × 3.0 μm^2^, after 15 min, 45 min and 90 min. The detailed vector PFM imaging parameters are listed in Table 1. Note that the actual contact resonance frequencies can shift due to changes of the tip-surface contact mechanics; hence, the measuring parameters (amplitude, frequency of the driving signal) needed to be frequently adjusted to keep the driving signals in the vicinity of the contact resonance frequency. Topographic images were processed using first-order line flattening to remove the effect of the surface tilt and thermal drift within the fast and slow scan directions. Cross-sectional profiles were averaged over 5 scan lines.

### Phase field simulation

The finite element phase field simulations for the domain evolution in the non-ergodic core (taken as a classical ferroelectric for simulation) and ergodic shell relaxor state were based on the models presented by Wang *et al*.^31^. In the simulations, the spontaneous polarisation and the electric potential were treated as degrees of freedom. In an ergodic relaxor, the phase field potential *H* included three terms: the electrostatic energy *H*^*ele*^, the Landau energy *H*^*lan*^ and the gradient energy *H*^*grad*^. The phase field potential *H* for the simulation was


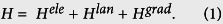


In the 2-dimensional case,


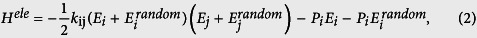



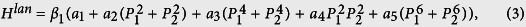



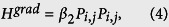


where *k*_*ij*_ is the dielectric tensor and *β*_*1*_ and *β*_*2*_ are the calibration coefficients, which depend on the domain wall energy and the domain wall width, respectively. In this expression, the Einstein notation was implied, in which the indices *i* and *j* ranged between 1 and 2. The Landau term *H*^*lan*^ contains an incomplete Taylor expansion up to the sixth order, and the parameters *a*_1_ to *a*_5_ are based on those used by Cross *et al*.^36^. The random field *E*_*i*_^*random*^ was assumed as Gaussian distribution miming the chemical disorder^37^; its strength was controlled by the variance of this distribution. Here, a moderate value of 8 kV/mm was adopted to insure that the random field was neither too strong to allow local domain switching nor too weak to show relaxor properties. The evolution of the order parameter P is determined by


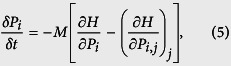


where *M* is a mobility parameter. For the simulation of the non-ergodic core, a model analogous to that used for the ergodic shell relaxors was used, but the random electric field was excluded. Thus, the potential could be written as


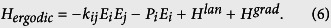


These models were implemented in the software FEAP^38^ using the finite element method. Based on the measured geometric dimensions of the core-shell microstructure and assuming that the region deeper than 500 nm from the surface was not affected by the biased-tip, a non-ergodic spherical core of radius 250 nm surrounded by an ergodic matrix of size 500 nm × 1,000 nm (depth × width) was simulated. A coherent interface was assumed between the core and the shell. Due to the rotational symmetry, the system could be reduced to a 2-dimensional configuration on the *x*-*z* plane for the simulation. The size of the finite elements should be fine enough to resolve the domain structure and to guarantee the precision of the results. Moreover, symmetric boundary conditions were set on the left and right edges of the box to ensure that the simulation represents a periodic case without the boundary effect. The reference potential at the bottom boundary was set to zero, and the electric field was assumed to undergo a linear potential decrease with distance between the top and bottom boundaries. The simulations revealed not only the polarisation state of the sample surface but also the domain configuration beneath the surface. For vertical poling, a +10 V *dc* voltage difference was applied, similar to the PFM experiment. A triangular potential *vs*. time distribution was employed for simulating the increase and the subsequent decrease of the electric field. The quasi-static simulation was performed with a potential increment of 0.1 V. Before poling, the equilibrium polarisation distribution in the non-ergodic core and the ergodic shell was awaited.

## Additional Information

**How to cite this article**: Liu, N. *et al*. Revealing the core-shell interactions of a giant strain relaxor ferroelectric 0.75Bi_1/2_Na_1/2_TiO_3_-0.25SrTiO_3_. *Sci. Rep*. **6**, 36910; doi: 10.1038/srep36910 (2016).

**Publisher’s note**: Springer Nature remains neutral with regard to jurisdictional claims in published maps and institutional affiliations.

## Supplementary Material

Supplementary Information

## Figures and Tables

**Figure 1:**
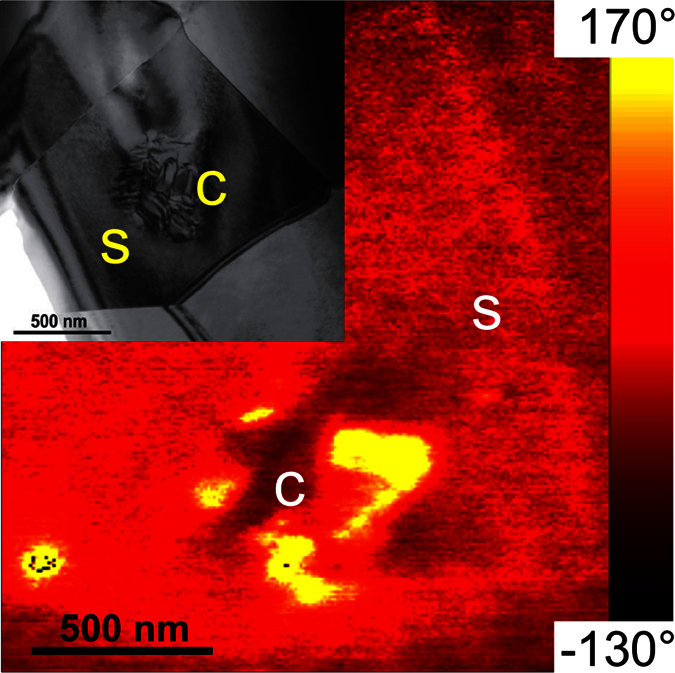
Core-shell structure of individual grains. PFM vertical phase image exhibiting distinct contrast in the core region. Inset: Typical TEM micrograph showing the core-shell microstructure (different location). The cores and the shells are marked with the letters C and S, respectively. Note that the contour of the grain is noticeable. TEM image reproduced with permission from ref. 1, © 2015 Wiley.

**Figure 2:**
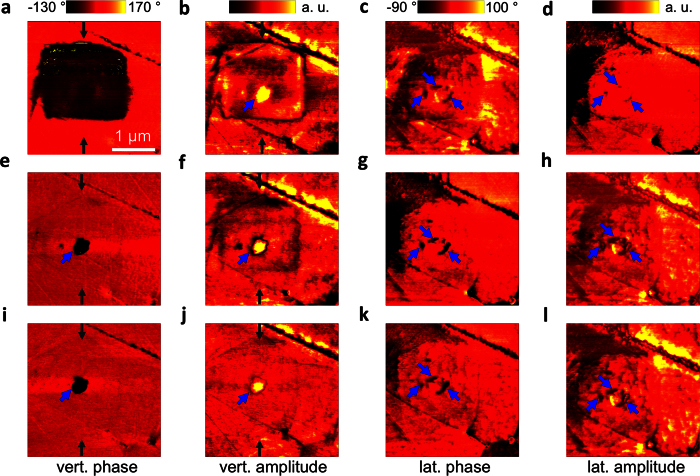
Evolution of the polarisation after poling the sample in a 1.5 × 1.5 μm^2^ region of interest, as indicated in Fig. S2m (Supplementary Information). (**a**–**d**) PFM images captured 15 min, (**e–h**) 45 min, and (**i–l**) 90 min after the completion of the poling experiment. The PFM data show the (**a**,**e**,**i**) vertical phase and (**b**,**f**,**j**) amplitude, as well as the (**c**,**g**,**k**) lateral phase and (**d**,**h**,**l**) amplitude. The blue arrows highlight core areas; the dark grey arrows indicate the locations where the cross-sectional profiles were drawn in Fig. S1 (Supplementary Information).

**Figure 3:**
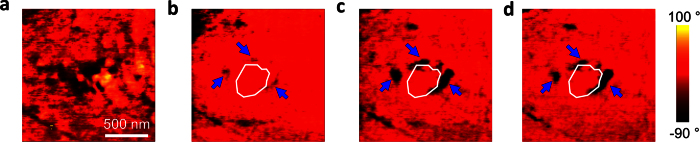
Evolution of the lateral domain at the core-shell interface after poling. (**a**) The virgin state of the lateral domain structure. (**b**) The electric field-induced lateral domain phase states 15 min, (**c**) 45 min, (**d**) 90 min after the completion of the poling experiment. The white line denotes the outline of the remanent vertical phase in the core after 45 min (Fig. 2e). The blue arrows highlight the newly formed lateral ferroelectric domain at the core-shell interface. Note: (**a–d**) are the enlarged images of the lateral PFM phase data from Fig. 2c,g,k and Supplementary Fig. S1n.

**Figure 4:**
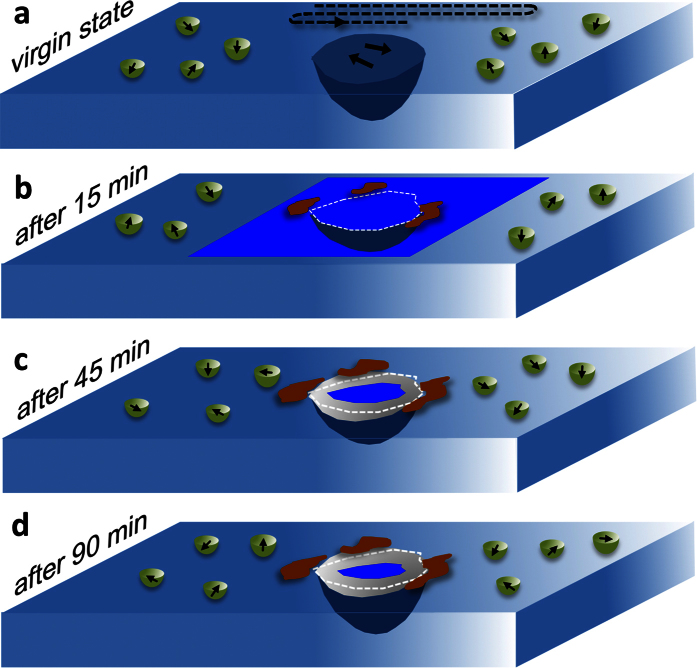
Scheme of the proposed model for the relaxation within the core-shell region of an individual grain. (**a–d**) Evolution of the vertical and the lateral domain configuration before (**a**) and after 15 min (**b**), 45 min (**c**) and 90 min (**d**) of vertical poling. The light blue planes represent a part of the Sr^2+^-rich shell, with the Sr^2+^-depleted core in the centre (dark blue hemisphere). The black arrows indicate the local domain configuration (polar nanoregions). The white dashed frames separate the tip-induced core from the shell. The dark blue area indicates a vertical downward-oriented domain and the grey area, a mixed vertical and lateral domain. The brownish areas indicate the possible lateral domain propagation from the interface to the shell. The dashed line in (**a**) illustrates the trace of the nanoscale line-by-line poling of an AFM tip.

**Figure 5:**
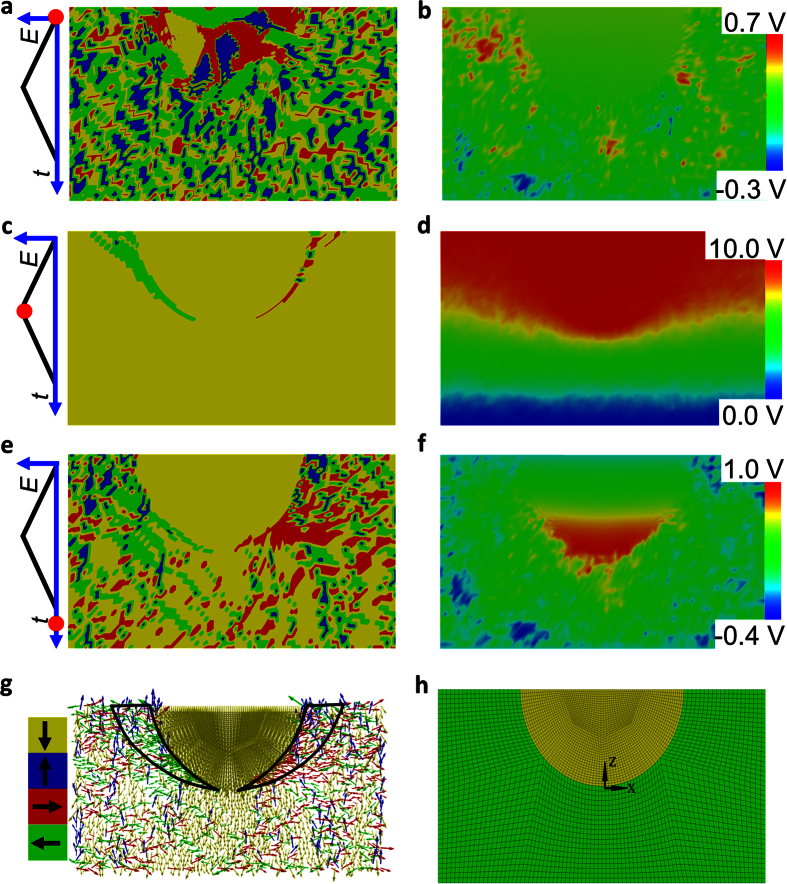
Phase field simulation of a formation of the field-induced polarisation. (**a,c,e,g**) Domain distribution and respective orientation of the polarisation. (**b,d,f**) Corresponding potential distribution. (**h**) Designed mesh in the *x*-*z* plane through the sample volume miming the geometry of a core-shell structure. The simulation evolves from the initial state (**a,b**) over a maximum potential state (**c,d**) to the remanent state (**e,f,g**). The detailed polarisation vector distribution at the remanent state is shown in (**g**). The marked regions (black frames) have a preferential lateral domain orientation. The colours in (**a,c,e**) and the arrows in (**g**) represent four polarisation orientations of the domains: yellow – down; blue – up; green – left; red – right. The length of the arrows indicates the magnitude of the polarisation vector. The three upper charts on the left illustrate the different stages of the simulation, in which red dots denote the current applied field and the *y*- and *x*-axis represent the electric potential and the time step, respectively.

**Table 1:** PFM imaging experimental parameters.

Cantilever	ASYELEC-02		AC240TM	
Direction	Vertical	Lateral	Vertical	Lateral
Driving amplitude (V)	3.0	2.5	3.5	3.5
Driving frequency (kHz)	926	1886	268	736
Scanning speed (μm/s)	3.1		2.3	
