# Supplementary material for: Revealing the core-shell interactions of a giant strain relaxor ferroelectric 0.75Bi1/2Na1/2TiO3-0.25SrTiO3
Source: Sci Rep. 2016 Nov 14;6:36910. doi: 10.1038/srep36910 (PMC5107923; doi:10.1038/srep36910)
Supplement: Supplementary Information [file srep36910-s1.pdf]

Supplementary Information

Revealing the core-shell interactions of a giant strain relaxor ferroelectric $0.75Bi_{1/2}Na_{1/2}TiO_3$-$0.25SrTiO_3$

Na Liu[1], Matias Acosta[2], Shuai Wang[3], Bai-Xiang Xu[3], Robert W. Stark[1], and Christian Dietz[1*]*

[1]*Institute of Materials Science and Center of Smart Interfaces, Physics of Surfaces, Technische Universität Darmstadt, Alarich-Weiss-Str. 10, 64287 Darmstadt, Germany*

[2]*Insititute of Materials Science, Nichtmetallische-Anorganische Werkstoffe, Technische Universität Darmstadt, Alarich-Weiss-Str. 2, 64287 Darmstadt, Germany*

[3]*Insititute of Materials Science, Mechanik funktionaler Materialien, Technische Universität Darmstadt, Jovanka-Bontschits-Str. 2, 64287 Darmstadt, Germany*

*Corresponding authors email addresses: stark@csi.tu-darmstadt.de, dietz@csi.tu-darmstadt.de.

Virgin state of the core-shell structure of $0.75Bi_{1/2}Na_{1/2}TiO_3$-$0.25SrTiO_3$

To identify surface regions with a distinct piezoresponse in the virgin state, we first investigated an untreated sample of $0.75Bi_{1/2}Na_{1/2}TiO_3$-$0.25SrTiO_3$ (abbreviated BNT-25ST) using vector piezoresponse force microscopy (PFM). Three different regions of interest (roi) were investigated (Fig. S1): roi 1: upper row Fig. S1a-f, roi 2: middle row Fig. S1g-l, and roi 3: bottom row Fig. S1m-r. In PFM, the amplitude and phase signals locally represent the magnitude of the electromechanical response and the domain orientation beneath the tip, respectively. It can be clearly seen from the lateral and vertical amplitude and phase signals (Fig. S1a-d, Fig. S1g-j and Fig. S1m-p) that these individual grains exhibit distinctive domain contrasts (blue arrows), whereas there is only a weak contrast in the surrounding areas. The presence of central and outer regions indicates the coexistence of two types of domain states. These states can be ascribed to the core-shell microstructure[1,2], which is related to the non-ergodic and ergodic relaxor states[3]. The observation of two distinct relaxor states within individual grains is in good agreement with transmission electron microscopy (TEM) study carried out on the same material[1]. The core in the topography images has a circular shape, whereas the outline in the lateral amplitude/phase images is asymmetric. Nevertheless, considering the outline to be an ellipse-like structure, the average size of the long and short axes from the three examples can be estimated to be 760 nm and 340 nm, respectively (Fig. S1). However, the core diameter of BNT-25ST, as estimated from the TEM results[3], was, on average, only 180 − 360 nm. There does not seem to be a sharp interface between the core and the shell[2], but the authors discussed a possible polarisation or stress mismatch at the interface[3,4]. Thus, the different sizes observed by TEM and PFM may be well explained by the different measurement and setup conditions (ambient conditions *vs*. vacuum, geometrical tip/surface convolution[5] *vs*. electron impact/distribution, electric field distribution from tip to sample *vs*. beam diameter).

The piezoresponse signal showed a stronger contrast in the lateral channels than in the vertical ones (*cf.* top and bottom row, Fig. S1). The domain configurations in the three regions of interest substantially differ. These differences can be attributed to individual grain orientations relative to the global coordinate system in the polycrystalline ceramic[6]. Hence, the ferroelectric domain orientation depends on the grain orientation and on the crystallographically allowed directions, in which the

crystal structure of the BNT-25ST is spontaneously polarized. The corresponding topography images of the three different regions of interest are shown in Fig. S1e, k and q. Interestingly, tiny hollows are detected in the core of all three locations. The hollows at roi 1, 2 and 3 are characterized by depths of approximately 1.2 nm, 0.8 nm and 0.9 nm, respectively (cross-section profiles drawn along the direction indicated by the grey arrows). The lateral dimensions of these hollows are 400 − 700 nm. The occurrence of the tiny hollows in the core is most likely caused by the macroscopic surface polishing or grinding. We speculate that the different chemical compositions of core and shell cause a distinct critical shear strength in both constituents, leading to selectively polishing-induced topographical hollows in the softer material[3]. Thus, the picture of the core embedded in the non-ergodic shell describes the situation well (Fig. 1a).

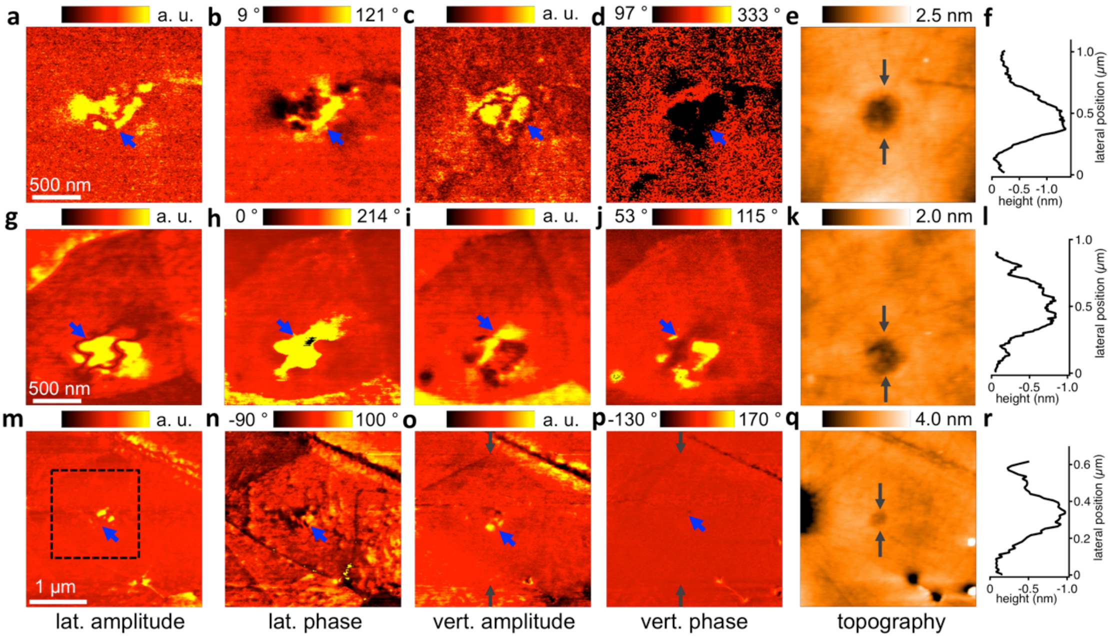

Figure S1. Visualisation of virgin domain states of core-shell microstructures at three different regions of interest. a-f, Region of interest 1. g-l, Region of interest 2. m-r, Region of interest 3. Each column shows the same PFM channel for the three regions of interest: (a,g,m) lateral amplitude and (b,h,n) phase, (c,i,o) vertical amplitude and (d,j,p) phase. The respective topography is shown in (e,k,q). The distinct domain structures in the core are revealed (blue arrows). At all three locations, the core is correlated with a depression in the topography, as illustrated in the cross-sectional

profiles (f,l,r) drawn along the dark grey arrows. The dashed black frame in (m) marks the 1.5 × 1.5 μm^2 that was poled (Fig. 2).

Evolution of the field-induced domain, as analyzed using cross-sectional profiles

To study the evolution of the field-induced domain and the relaxation behaviour in the core-shell region, averaged cross-sectional profiles taken in the vertical phase (Fig. S1p, Fig. 2a,e,i), amplitude (Fig. S1o, Fig. 2b,f,j) and the respective topography are displayed in Fig. S2. The grey box in Fig. S2a highlights the diameter (approx. 380 nm) of the core, as determined from the topography image. After preparation, the core was initially 0.8 nm deep (red line). However, 15 min after poling, the remaining depth was reduced to 0.2 nm (blue line). Then, the depth increased again to 0.3 nm and 0.5 nm after 45 min (black line) and 90 min (grey line), respectively, whereas the diameter of the structure remained constant. From the cross-sectional profiles, the following information can be extracted. For the virgin state, the vertical phase (Fig. S2b, red line) is constant across the core-shell region and the corresponding amplitude (Fig. S2c, red line) exhibits only a very small magnitude, suggesting a weak piezoresponse in the vertical direction. This is in agreement with the data in Fig. S1m-p, where a weak vertical and strong lateral piezoresponsive region was observed in the non-ergodic core area. 15 min after the poling, the phase shift in the poled area was –150° (Fig. S2b, blue line), indicating that a long-range ferroelectric domain, whose polarization vector points downward, has developed. The poled area is approx. 2 µm large. The corresponding vertical amplitude response (Fig. S2c, blue line), however, was only affected over a lateral width smaller than 380 nm instead of the fully poled distance of 2 µm. This observation and the evolution of the vertical amplitude (Fig. 2) imply that the induced vertical domain in the ergodic shell partially relaxed within the first 15 min. Then, 30 min later (Fig. S2b, black line), the diameter of the induced vertical domain (characterized by 150° in the phase) decreased from initially 2 µm to 380 nm and then remained stable (Fig. S2b, grey line), *i.e.*, the poled area shrank down to the core. This means that an irreversible transformation from the non-ergodic relaxor state to the ferroelectric state can occur in the core, whereas the transformation from the ergodic state to the ferroelectric state is reversible within the shell[3]. We also observed that the amplitude response of the field-induced domain

region further laterally shrank in size, accompanied by the propagation and growth of the in-plane polarization at the core-shell interface.

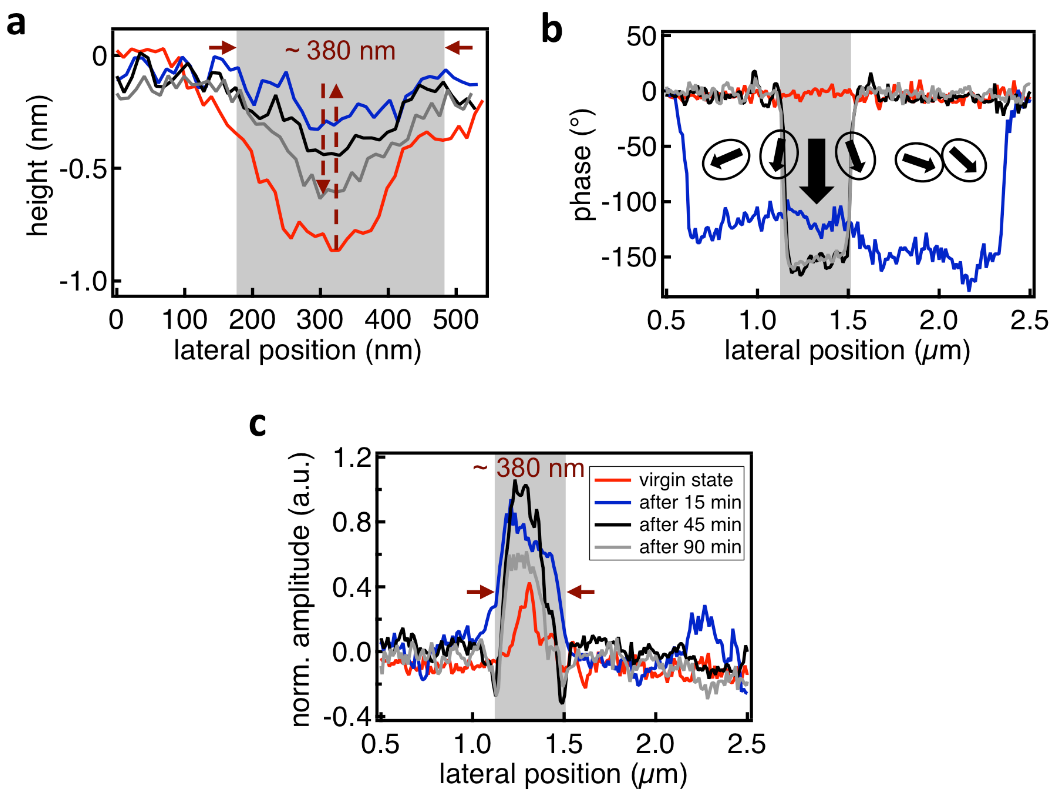

Figure S2. Evolution of the field-induced vertical domain and its relaxation behaviour, as well as the corresponding topography changes at the core-shell region. a, Cross-sectional profiles of the topography before and after poling, drawn along the arrows in Fig. S1q. b, Cross-sectional profiles of the vertical phase before and after poling, drawn along the arrows in Fig. S1p, and Fig. 2a,e,i from the main article. c, Cross-sectional profiles of vertical amplitude before and after poling, drawn along the arrows in Fig. S1o and Fig. 2b,f,j from the main article. The virgin state in each image is indicated by the red solid line, whereas the blue, black, and grey solid lines indicate the signals 15 min, 45 min and 90 min after poling, respectively. The grey boxes highlight the position of the core, which is ~ 380 nm in width, showing the topographical hollows and polarization states of the tip-induced vertical domain. The red upward and downward arrows (a) guide the reader through the evolution of the topography at the core after the poling experiment. Immediately after poling, the hollow nearly disappeared (upward arrow, a) and grew back with time (downward arrow, a). The black arrows (b) mime the potential domain configuration (polar nanoregions) at the sample surface.

References

1. Acosta, M., Jo, W. & Rodel, J. Temperature- and frequency-dependent properties of the $0.75Bi_{1/2}Na_{1/2}TiO_3$-$0.25SrTiO_3$ lead-free incipient piezoceramic. *J. Am. Ceram. Soc.* **97,** 1937–1943 (2014).

2. Koruza, J., Rojas, V., Molina-Luna, L., Kunz, U., Duerrschnabel, M., *et al.* Formation of the core-shell microstructure in lead-free $Bi_{1/2}Na_{1/2}TiO_3$-$SrTiO_3$ piezoceramics and its influence on the electromechanical properties. *J. Eur. Ceram. Soc.* **36,** 1009–1016 (2016).

3. Acosta, M., Schmitt, L. A., Molina-Luna, L., Scherrer, M. C., Brilz, M., *et al.* Core-shell lead-free piezoelectric ceramics: Current status and advanced characterization of the $Bi_{1/2}Na_{1/2}TiO_3$-$SrTiO_3$ system. *J. Am. Ceram. Soc.* **98,** 3405–3422 (2015).

4. Park, Y., Kim, Y. H. & Kim, H. G. The effect of grain size on dielectric behavior of $BaTiO_3$ based X7R materials. *Mater. Lett.* **28,** 101–106 (1996).

5. Villarrubia, J. S. Algorithms for scanned probe microscope image simulation, surface reconstruction, and tip estimation. *J. Res. Natl. Inst. Stan.* **102,** 425–454 (1997).

6. Uchino, K. *Advanced piezoelectric materials* (USA, 2010).
